# Health IT and digital health: The future of health technology is diverse

**Published:** 2018-09-08

**Authors:** Bertalan Mesko

**Affiliations:** ^1^ The Medical Futurist Institute, Budapest, Hungary; ^2^ Semmelweis University, Department of Behavioral Sciences, Budapest, Hungary

## 1. The evolution of medical technology

Physicians used to make informed decisions with a very limited set of tools but a growing amount of experience and knowledge that spans generations. With the birth of modern medicine in the 18th century, the practice of medicine has gradually become dependent on the use of technology. It started with simpler methods, hollow wooden tubes (the first stethoscope) and X-rays, and evolved into cloud-based algorithms and virtual reality. By the end of the 20th century and the beginning of the 21st century, medicine has become a technological profession. Without it, no physician can be up-to-date, make informed decisions, or be able to legally practice medicine. Technologies have been infused into the delivery of care too.

When personal computers became widely available in the 1990s, e-health emerged [1]. When such computers could be connected to networks, telemedical services appeared [2]. The rise of social media networks made room for medicine 2.0 and health 2.0 [3]; while the advent of mobile phones and later smart-phones summoned mobile health [4]. But in the 2010s, the rate at which disruptive technologies appear is becoming overwhelming for both patients and their caregivers [5].

As patients started to gain access to information and technologies that before were only available in the so-called 'ivory tower of medicine,' patient empowerment was born. This made patients proactive and wish to have an equal-level partnership instead of a hierarchical dependence on their physicians. They want to take part in the decision-making process regarding their health and contribute data they measure at home.

This cultural transformation that changes the essence of the doctor-patient relationship and the basics of healthcare is called digital health. We define digital health as *"the cultural transformation of how disruptive technologies that provide digital and objective data accessible to both caregivers and patients leads to an equal-level doctor-patient relationship with shared decision-making and the democratization of care."*

## 2. Health IT and digital health

**Figure 1:**
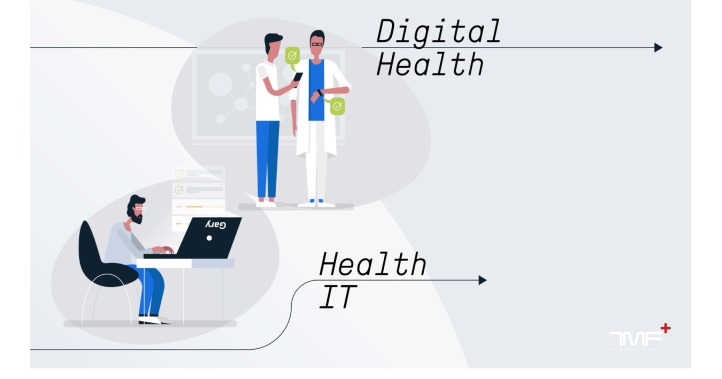
The distinction between health IT and digital health

When discussing the future of technologies in healthcare, one must make a clear distinction between issues regarding IT (Information technology) and digital health. The two areas are often intermingled while their nature and the solution they require are different on many scales. In a nutshell, IT issues impact physicians' everyday job the most but can be dealt with in the short term. Digital health has more impact on cultural changes and entails a long-term process (Figure 1).

To give some examples of IT issues: anti-virus software started running on a computer that monitored a patient who was undergoing heart surgery [6]. The monitoring equipment failed during the operation. An investigation by the Food and Drug Administration (FDA) found that anti-malware software was responsible for the failure of the equipment, as it was set to scan for viruses every hour, against the recommendation of the equip-ment maker. This problem can be instantly resolved by turning off the scanning function of the software.

In another example, many electronic medical records systems do not communicate with each other, so accessing the required information to make a medical decision can become chal-lenging for physicians dealing with multi-institutionalized pa-tients. Such issues can be resolved by developing computer pro-grams that can communicate between the different electronic health records software packages and retrieve patient data for direct access by the physicians. Lastly, during the WannaCryscandal, a global cyberattack infected 300,000 computers in 150 countries using hacking tools [7]. It also crippled the National Health Service (NHS) in the United Kingdom. UK hospitals were shut down and had to turn away non-emergency patients after ransomware ransacked its networks. Since that attack, hos-pitals doubled down on cybersecurity.

Digital health issues are different in nature. Patients bring data they measure with sensors for their health or medical condition to the doctor-patient meeting and expect their caregivers to address technological questions in addition to medical questions. A medical robot can be a valuable asset to the team in a healthcare facility to deal with the labor burden during night shifts, when fewer people are working the floors. While the robot can facilitate the caregivers' job, it takes time and effort to get accustomed to a robot being a member of their team. Medical professionals use technologies daily as medical records are being digitized worldwide and smartphone apps are widespread.Since the dawn of digital health, medical professionals have gradually had to accommodate health sensors and internet-based services. Using digital health is a team effort, thus the era of lonely doctor heroes will end. The success of providing care depends on collaboration, empathy, and shared decision making. What is needed for implementation of care in the digital era is a newly defined cooperation between patients and their caregivers that allows room for new technologies. Nevertheless, a well-functioning patient-physician relationship will remain an essential part of healing. One seminal study revealed that the empathy skills of physicians can influence diabetic patients' objective clinical chemistry outcomes, the incidence of complications, and subjective well-being [8].

## 3. Framework for shifting between health IT and digi-tal health

The distinction between IT and digital health and the imple-mentation of digital health into practice require new frameworks. As digital health makes patients the point-of-care, a new status quo and new roles for both patients and caregivers are surfac-ing that heavily affect healthcare policies and shape the digital health framework. While constructing the digital health frame-work in terms of regulatory policies, several important aspects should be taken into account. Policy makers are expected to make every new technology available quickly, otherwise con-sumers may start using the technology without the proper reg-ulations in place. The wearenotwaiting initiative is a perfect example for this kind of pressure [9]. As there was no single device on the market to monitor blood sugar and supply insulin automatically, creative patients invented a do-it-yourself version from existing technologies. A movement grew out of the initia-tive and campaigned for the market introduction of an 'artificial pancreas' for years. One of the leading figures of the movement, Dana Lewis, used the device for almost two years before the FDA finally approved it.

Although the artificial pancreas was ultimately a success, such (social) initiatives come with risks too. Medical technologies including surgical robots, pacemakers, and insulin pumps have been shown to be prone to hacking. Health sensors used by patients at home might not be accurate and lack an evidence-based foundation. Patients might find misleading information online that leads to erroneous self-diagnosis.

Policy makers should therefore find ways to promote the safe use of digital health technologies, regulate them as fast as possible, and keep patients' data safe.

## 4. Examples for the implementation of digital health

Four categorical examples are described below that illustrate how digital health can be implemented into a novel framework. These examples entail patient centricity, regulating disruptive technologies, preventing ethical challenges, and promoting the use of digital health [10].

In terms of **putting patients at the center of healthcare,** the creation of the "Patients Included" badge is an exemplary initiative. The badge helps to identify medical events where patients are either among the speakers or involved in the organizing committee. The concept was developed at an innovation hub called the REshape Center of the Radboud University Medical Center in 2010. Events such as Stanford Medicine X and Doctors 2.0, and You even launched e-patient ambassador programs and invited patients to speak. The British Medical Journal was awarded a special "Patients Included" certificate to acknowledge and encourage their involvement of patients in medical publishing.

With respect to **regulating disruptive technologies,** the FDA cleared AliveCor's smartphone ECG, which is available for both Apple and Android phones, to be used by patients. It was the first digital health sensor to receive clearance. AliveCor also received clearance for an algorithm that allows the smartphone ECG app to detect atrial fibrillation. In 2017, the FDA cleared AliveCor's Kardiaband ECG reader as the first medical device accessory for the Apple Watch. These developments pave the way for additional approvals for digital health sensors that will become available to patients.

**Regulators must prevent ethical pitfalls** when shaping the digital health framework. With the advent of do-it-yourself gene therapies that attempt to modify one's genomic material, the FDA acknowledged that gene therapy products and "do it yourself" kits intended for self-administration are available to the public, but stressed that the sale of these products is against the law. The FDA's public stance on this issue and regulatory follow-up on the one hand cautioned consumers about the inher-ent dangers of self-administered gene therapy and, on the other hand, ensured that these therapies have either been approved by the FDA or are being studied with appropriate regulatory over-sight.

One of the hardest challenges in the framework shift is **cre-ating regulations that promote the use of digital health** without pushing stakeholders to do something that is against their will. In a successful attempt to consolidate digital health with patient care, the NHS rolled out a program that encourages physicians to prescribe apps to patients with a chronic condition. A study found that digital health helped reduce the number of patient visits by 25%. By curating reliable medical apps, primary care physicians will be asked to recommend apps that are free or cheap in an attempt to give patients more power and reduce visits to doctors.

## 5. Conclusion and introducing the "Gary-rule"

To help medical professionals and policy makers make a clear distinction between health IT and digital health whenever they need, a general rule of thumb, the "Gary-rule" might come handy. If a technological issue comes up in a healthcare setting such as the antivirus software becomes outdated or the electronic medical record system stopped working and we have to call Gary, the IT guy, as he is alone capable of solving it whatever methods he uses, it's an IT issue.

If Gary is not enough to solve the issue because more stakeholders of healthcare must get involved, (e.g. letting patients to bring the data of their trackers into the practice and merging that with electronic medical records; or allowing physicians to do remote consultations on a regular basis), it's digital health.

It's hard to draw a definitive line between health IT and digital health issues (Table 1), although drawing a territory between them might help caregivers use new technologies to improve and expedite their job so that in the end they may spend more time with patients.

**Table 1:**
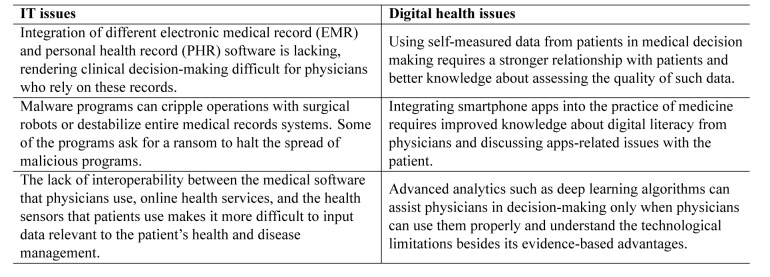
Examples of IT and digital health issues.
